# Privacy-preserving decentralized learning methods for biomedical applications

**DOI:** 10.1016/j.csbj.2024.08.024

**Published:** 2024-08-30

**Authors:** Mohammad Tajabadi, Roman Martin, Dominik Heider

**Affiliations:** a Institute of Computer Science, Heinrich-Heine-University Duesseldorf, Graf-Adolf-Str. 63, Duesseldorf, 40215, North Rhine-Westphalia, Germany; b Center for Digital Medicine, Heinrich-Heine-University Duesseldorf, Moorenstr. 5, Duesseldorf, 40215, North Rhine-Westphalia, Germany

**Keywords:** Federated learning, Split learning, Swarm learning, Gossip learning, Edge learning

## Abstract

In recent years, decentralized machine learning has emerged as a significant advancement in biomedical applications, offering robust solutions for data privacy, security, and collaboration across diverse healthcare environments. In this review, we examine various decentralized learning methodologies, including federated learning, split learning, swarm learning, gossip learning, edge learning, and some of their applications in the biomedical field. We delve into the underlying principles, network topologies, and communication strategies of each approach, highlighting their advantages and limitations. Ultimately, the selection of a suitable method should be based on specific needs, infrastructures, and computational capabilities.

## 1 Introduction

The recent developments in artificial intelligence (AI) have encouraged various research disciplines to adopt AI methods progressively. Modern breakthrough achievements such as ChatGPT [1] or AlphaFold [2] bring a new drive into this development, encouraging new and more substantial development and adaptation of AI for specific fields. In particular, the application of AI in the biomedical field has enormous potential to change medicine in the future. The effects begin with a high-specific analysis of the development or repurposing of drugs [3] and reach massive epidemiological modeling [4]. From a long-term perspective, new treatments, prognostics, or monitoring methods can significantly improve the quality of medicine for patients and medical staff [5], [6], [7]. However, especially in the biomedical field, the application and development of AI models are complicated and raise various concerns regarding privacy, data security, regulations, and data responsibilities [8], [9]. In contrast to other fields, biomedical data are, by nature, highly sensitive. For instance, the European General Data Protection Regulation (GDPR) outlines general obligations for data processing, which constrain the sharing of private data, including medical patient data. Therefore, decentralized learning can be seen as a key success factor for artificial intelligence in healthcare, offering a technical solution that effectively supports privacy-by-design [10].

Handling sensitive medical data causes a direct conflict in creating new AI models based on these patient's data. The power of generalized and accurate AI models relies on the availability of massive amounts of data, which is often rare at one site, such as a single hospital. While the traditional machine learning concepts rely on a centralized, accessible data location, the decentralized learning methodologies allow us to break through these limitations by providing techniques to build new AI models without sharing the patients' sensitive data [11].

Over the last decade, multiple concepts have been introduced to address these privacy concerns. Methods such as gossip, federated, split, swarm, and edge learning were established and have certain methodical overlaps but also differences. This review will break down the different concepts and give examples of current applications in biomedical research, including the ups and downsides of these methods.

## 2 Overview and terminology

In contrast to centralized and traditional machine learning, decentralized learning covers algorithms and implementations, which rely on at least two participants performing their model training primarily independently and sharing their models or parameters afterward. We differentiate between centralized and decentralized approaches based on where data is stored.

**Fig. 1:**
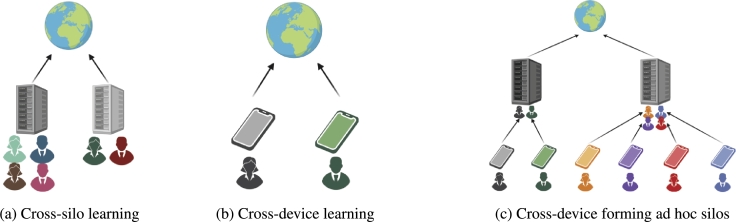
Various settings for decentralized learning. a) shows a cross-silo setting, which is typical for federated learning as assuming they have data from multiple sources such as patients. b) presents a typical cross-device setting based on, for example, smartphones contributing data for a globalized model, while c) illustrates a hybrid setting, combining cross-device and cross-silo.

Currently, there are two established settings for decentralized learning: cross-device (Fig. 1a) and cross-silo (Fig. 1b) [12]. The main difference lies in the number and type of data sources and devices involved. In decentralized learning, data is either stored in silos, which are institutional entities such as companies or hospitals containing data from multiple sources like different patients or on small devices (cross-device) like smartphones or IoT (Internet of Things) devices, which typically hold data from a single origin.

This distinction also affects computational power since most smartphones and IoT devices are limited in their ability to perform heavy computations, such as those required for machine learning. Despite these differences, the two settings can be combined in nested or hierarchical implementations (Fig. 1c). For example, devices can form ad hoc silos, acting as intermediaries as described in Yang's hierarchical federated learning framework [13].

**Fig. 2:**
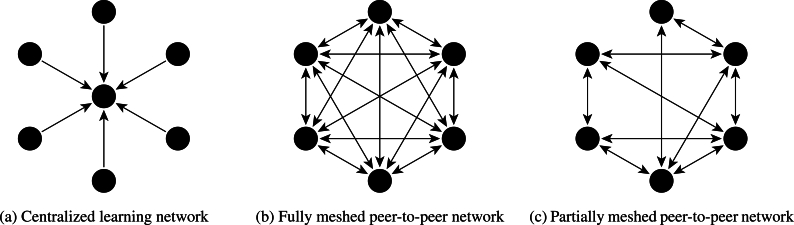
Different network topologies for decentralized learning approaches. a) illustrates a centralized scheme used in federated learning, split learning, and edge learning, b) presents a fully meshed peer-to-peer network that is used in swarm learning, and c) shows a partially meshed network that is common for a random walk within a gossip learning network.

Another aspect that varies between different decentralized learning approaches is the network topology employed. Devices or organizations may form a centralized network (Fig. 2a), where a central server manages the learning process. Alternatively, the central server can be removed, allowing nodes to communicate directly in a fully meshed peer-to-peer network (Fig. 2b), where every node is connected to all others. In this case, all participants in the network need to know each other, which is reasonable for cross-silo implementations (Fig. 1b), e.g., hospitals. A third option is a partially meshed peer-to-peer network (Fig. 2c), in which not all nodes are directly connected, and it is suitable for cross-device (Fig. 1a) implementations due to the larger and more dynamic nature of the network.

In federated learning and split learning, nodes communicate with a central server within a centralized network. These approaches are suitable for both cross-silo and cross-device applications. Swarm learning, on the other hand, uses a fully meshed peer-to-peer network and is better suited for cross-silo scenarios where a few organizations collaborate to build machine learning models. Gossip learning operates in a partially meshed peer-to-peer network and is more appropriate for cross-device applications. Lastly, edge learning often employs a centralized scheme and is primarily associated with cross-device applications.

In the following sections, we review different decentralized learning methods used in the biomedical field and discuss their advantages and disadvantages.

## 3 Gossip learning

In 2013, Ormándi et al. [14] published their manuscript about gossip learning, introducing a partially meshed peer-to-peer network providing a privacy-aware, decentralized approach to perform machine learning with linear models over multiple peers. In gossip learning, not every peer is communicating with each other. Instead, multiple random walks are performed over the partial peer-to-peer network, averaging each round of the model's coefficients until all peers contain the final values. This decentralized machine learning concept was named gossip learning as an analogy to gossip, which is often randomly shared among people. As a result, it is entirely decentralized but technically inefficient since information must be shared and averaged multiple times. In the initial manuscript, the model performance converged, depending on the failure rates and dataset, after several hundreds or thousands of cycles [14].

Multiple publications showcased how gossip learning could be used for image classification based on deep neural networks on iid (independent and identically distributed) and non-iid data [15], [16]. In 2023, Chen et al. demonstrated gossip learning for brain tumor segmentation on multi-parametric MRI [17]. While gossip learning was one of the first decentralized learning methods introduced, it never gained as much attention as federated learning, especially in biomedical applications.

## 4 Federated learning

Federated learning, introduced by McMahan et al. [18] in 2016, is a type of decentralized learning in which several nodes collaboratively train a machine learning model without sharing any private data. In federated learning, private data stays local, and only the model parameters are shared with a central server (Fig. 2a).

A federated learning system has two primary roles: clients and the server. Clients (or workers) conduct local training on their datasets, preserving privacy and reducing central data aggregation. The server coordinates the learning process, aggregating locally trained models and updating the global model. It periodically combines models from clients and distributes the updated model back until convergence.

Although federated learning was initially developed for neural networks, it has since been adapted to include other machine learning methods such as logistic regression and tree-based models, expanding its range of applications. Numerous federated learning applications using global patient data have been published, as systematically reviewed by Crowson et al. [19]. The majority of those studies evaluated imaging results and performed binary classification. Research into data protection and privacy concerns related to federated learning has been addressed in several studies [20], [21]. Additionally, Torkzadehmahani et al. [22] reviewed various privacy-preserving techniques in the medical field, including differential privacy [23], homomorphic encryption, secure multi-party computation [24], federated learning, and hybrid approaches. While most publications on federated learning focus on a traditional centralized scheme, some explore peer-to-peer network implementations.

In 2018, Lee et al. [25] developed and evaluated a federated learning platform for patient similarity across multiple clinical institutions. Each institution computed local statistics on their Electronic Health Record (EHR) data, creating binary hash codes for patients. These codes enabled efficient similarity searches using Hamming distance, with privacy protected by homomorphic encryption. The central server aggregated these encrypted results to form a global patient similarity model. This framework allowed sophisticated analyses without sharing raw data, ensuring privacy and enabling accurate disease incidence predictions for cross-institutional studies. Validation through similarity searches for various disorders confirmed the approach's feasibility and effectiveness, creating a robust, privacy-preserving platform for patient similarity analysis.

In 2022, Hauschild et al. [26] developed Federated Random Forest (FRF), demonstrating its effectiveness for various healthcare applications. They tested FRF on datasets for liver disease prediction, hepatocellular carcinoma diagnosis, breast cancer classification, lung tumor gene expression, and lung cancer classification. The authors proved that FRF achieved results comparable to those of centralized models. FRF also proved more stable and outperformed local models with imbalanced datasets, a common healthcare challenge. This study showed that FRF could achieve high performance through a collaborative federated learning approach, even with partial data on each client. Similarly, in 2022, Gencturk et al. [27] introduced a Boosting-based Federated Random Forest (BORF) algorithm tailored for horizontally partitioned data in a federated learning environment. Evaluated on four healthcare datasets, BOFRF enhances predictive performance, particularly for sites with imbalanced data. Current developments indicate FRF's potential for patient subtyping and improved cluster interpretability [28].

Moreover, in 2023, Pfeifer et al. [29] released Ensemble-GNN, a federated graph neural network for patient classification. This model is trained on protein-protein interaction (PPI) network data for breast cancer patients, incorporating genomic characteristics and functional protein relationships. Ensemble-GNN decomposes the PPI network into subgraphs, trains multiple GNN models on these, and combines them into a global federated classifier using GNNSubNet [30] to infer relevant network communities. In non-federated settings, Ensemble-GNN achieved accuracy comparable to Random Forest classifiers, while in federated settings, it outperformed local models.

Furthermore, the extensive body of federated learning research has culminated in the development of FeatureCloud. This user-friendly and comprehensive platform offers a range of combinable software modules through an app store to facilitate federated learning [31].

## 5 Split learning

In 2018, Gupta et al. [32] introduced split learning, a form of decentralized learning aimed at training deep neural networks over multiple agents without sharing private data. In this method, each party holds certain lower layers of the network while the server manages the remaining layers. In the simplest form with a single node and a server, the node starts training by forwarding the input through its layers up to a cut layer and sends the output of that layer, or smashed data [33], along with labels to the server. The server passes this data through its layers, computes the loss, propagates the loss back through its network, and then sends gradients back to the node to update weights. This process repeats until the model converges. For multiple nodes, training occurs in a round-robin fashion, with the server sending the last trained model to the next node. To avoid sending labels to the server, the authors proposed a U-shaped method, where the network is wrapped around its end layers. In this way, the server sends the parameters of its final layer back to the node where the loss is calculated. The authors of the paper demonstrated that split learning significantly reduces the amount of data transferred compared to federated learning [32].

Given its efficiency and privacy-preserving benefits, split learning has been increasingly adopted in healthcare applications. In 2023, Li et al. employed the concept of split learning for medical image classification and clinical concept predictions from structured EHR data [33]. For the image classification tasks, they used Convolutional Neural Networks (CNNs), and for the EHR data, they used Transformers and Fully Connected Neural Networks (FCNNs). They compared their results with those of federated learning and centralized learning and showed that while the accuracies of the models were similar, the number of parameters forwarded in the network was considerably lower in the split learning setting, thereby improving efficiency and lowering computational costs.

To address the computation limitations in Internet of Medical Things (IoMT) devices, Ni et al. [34], in 2024, proposed federated split learning (FedSL) for resource-constrained wearable IoMT devices. Their model consists of two phases: first, a feedforward and backpropagation process is performed. Then, the devices upload their updated subnetwork parameters to the server for aggregation, which are then sent back to the devices. They simulated a network with five devices and used ResNet-18 to classify chest X-ray and optical coherence tomography (OCT) images under IID (Independent and Identically Distributed) and non-IID settings. Their results show that FedSL slightly outperforms traditional federated learning.

In 2022, Zhang et al. [35] introduced SplitAVG for heterogeneous medical imaging. In this approach, the server concatenates the smashed data (or feature maps) from all participants and feeds the combined parameters to its portion of the model. After computing the loss, the server backpropagates the gradients through the network and back to the participants. Subsequently, both the server and the participants update their weights. The authors highlight that SplitAVG can capture more abundant and unbiased information when feature maps from local institutions are concatenated at earlier layers. Consequently, they selected the first convolutional layer as the cut layer in their experiments with ResNet-34 and ResNet-50. Experimental results on synthetic and real-world medical image datasets demonstrated that SplitAVG outperforms traditional federated learning methods in scenarios with high data heterogeneity.

In a similar work, in 2022, Joshi et al. [36] proposed Multi-Head Split Learning (MHSL) in which the clients' models (parameters) are concatenated at the end of each forward path, thereby eliminating the need for client model synchronization after each round.

In 2022, Ayad et al. [37] presented modifications to the original split learning system for Electrocardiography (ECG) classification on resource-limited edge devices. By incorporating an autoencoder to reduce data transfer and an adaptive threshold mechanism to minimize updates during back-propagation, they decreased communication and computation overhead with minimal performance loss.

## 6 Swarm learning

In 2021, Warnat-Herresthal et al. [38] introduced the term swarm learning to describe a specific approach to peer-to-peer collaborative machine learning. Prior to this, particle swarm optimization (PSO) [39] was a well-known heuristic method for optimizing a problem by searching for an optimal solution in a solution space. Although some authors in the literature have occasionally used the term swarm learning to refer to PSO [40], [41], [42], [43], [44], [45], [46], it is now more consistently applied to a decentralized form of collaborative learning. In this context, swarm learning involves multiple parties, each with their own local datasets, collaborating through a fully meshed peer-to-peer network without the need for a central server. This approach is particularly suited for cross-silo settings, where data is distributed across different organizations. Like other types of collaborative learning, this decentralized structure allows for the development of a machine learning model that utilizes the diverse data and computational resources of the participants, resulting in a model that is more robust and accurate than those developed individually.

Warnat-Herresthal et al. [38] used the swarm learning network to develop classifiers for four diseases, namely, COVID-19, tuberculosis, leukaemia, and lung pathologies. They simulated a swarm learning network by dividing datasets among multiple nodes and performing collaborative training. Through extensive simulations using multiple datasets, they showed that the swarm learning framework outperforms individual models trained locally at the nodes.

In 2021, Fan et al. [47] explored group fairness in swarm learning in medical settings. In the context of group fairness, as opposed to device-level fairness [48], [49] and collaborative fairness [50], [51], the goal is to create systems that avoid discrimination against particular groups. They implemented swarm learning for skin lesion classification, and they used sex and age to examine the bias. They simulated four institutions by dividing the data into four subgroups, namely, Male and Age ≥60, Male and Age <60, Female and Age ≥60, and Female and Age <60. Then they trained a VGG19 [52] network for three scenarios: swarm, centralized, and individual learning. Their reports show that while swarm learning achieves better performance compared to individual training, it does not amplify biases compared to the centralized model.

Saldanha et al. [53], in 2022, developed a swarm learning pipeline to predict molecular alterations from histopathology images. They used three datasets stored on separate servers and employed ResNet-18 for training in three scenarios: local, swarm, and centralized. Their results demonstrated that the swarm learning framework could predict BRAF mutational status and microsatellite instability (MSI) with performance comparable to the centralized model and superior to local models.

In 2023, Shashank et al. [54] employed the swarm learning framework for breast cancer tumor diagnosis. They ran their simulation with two nodes and showed that the accuracy of the models using swarm learning was slightly better than the local models.

In another study, in 2023, Mohammed et al. [55] simulated a swarm learning network with three nodes for diagnosing diseases in human nails. Their experiments used various versions of the dataset, differing in the number of disease classes and data distributions (both skewed and equal). They employed transfer learning models, specifically employing VGG16 and InceptionV3 as source models, for prediction. The results showed that while the swarm learning models performed well, the centralized model still outperformed them.

## 7 Edge learning

Edge learning is another concept within the context of decentralized learning. In edge learning, an edge refers to either an edge device or an edge server. Edge devices are processing units located at the periphery of the network, such as smartphones, cameras, or laptops (cross-device). These devices are usually the data storing devices. Edge servers, on the other hand, serve as intermediaries with the shortest path to the edge devices and act as gateways to a larger network. These edge servers possess greater computational power compared to edge devices.

Edge learning is built upon the concept of edge computing as opposed to cloud computing. Edge computing involves performing computations as near to the data sources as possible, rather than at the cloud. The reason is that transferring raw data to cloud servers raises communication costs, delays system responses, and compromises data privacy [56]. The focus of edge learning, therefore, lies in addressing issues related to network latency, bandwidth, the processing power of devices, and other network-related challenges. For instance, Ye et al. proposed EdgeFed [57] to optimize federated learning based on the concept of edge computing. In this approach, computation is offloaded by assigning mobile devices the task of training lower layers, while more computationally intensive tasks are assigned to edge servers with greater computational power. EdgeFed also leverages the typically higher bandwidth between clients and the edge server compared to that between the edge server and the central server to reduce global communication costs compared to FedAvg.

In the medical field, the concept of edge learning is entangled with the IoMT which is a network of Internet-connected medical devices to collect, manage, and process healthcare data. In 2021, Rahman et al. [58] developed an edge IoMT system that employs state-of-the-art deep learning applications to detect a wide range of COVID-19-related symptoms. By processing data locally using edge GPUs, the system ensures that health data remains within the user's environment, thereby enhancing privacy and minimizing latency.

Priyadarshini et al. [59], in 2018, proposed a healthcare monitoring system called DeepFog, which comprises three layers: the physical layer, the fog layer, and the cloud layer. The physical layer includes edge devices, such as sensor-equipped devices and smartphones, that collect data. The fog layer consists of edge servers, while the cloud layer encompasses cloud servers and data stations. The core concept is that data collected by edge devices is not sent directly to the cloud. Rather, it is transferred to the edge server, where deep learning frameworks are employed for stress type categorization, diabetes prediction, and hypertension risk assessment. These data are shared with domain experts, doctors, and healthcare professionals who can take precautionary measures and immediate action in emergency situations. This approach prevents overloading the cloud server with data and prediction tasks by offloading learning tasks to the network's edge.

In 2019, Queralta et al. [60] introduced a health monitoring system leveraging edge computing. Their architecture comprises five layers: the sensor layer (wearable devices), edge layer (smart edge gateways), fog layer (Low Power Wide Area Networks), cloud layer, and application layer (end-user terminals). In their framework, sensor nodes gather medical data and transmit it to edge gateways for initial processing and developing machine learning models. Subsequently, results are forwarded to low-power access points for further processing before reaching the cloud for final analysis. This architecture aims to alleviate computational burdens on sensor nodes by offloading intensive tasks to edge gateways. The authors validate their approach by implementing a recurrent neural network (RNN) for human fall detection.

Similarly, in other studies, the authors developed a hierarchical architecture known as HiCH [61], [62], in 2017 and 2018, which leverages both edge and cloud computing for medical applications. The HiCH architecture described in [61] assigns training tasks to the cloud and inference tasks to edge servers. This approach allows heavy computational training to be handled by the cloud while inference tasks are executed at the edge for quicker decision-making. To validate their system, they implemented a Support Vector Machine (SVM) for detecting cardiovascular diseases. In [62], the HiCH architecture is employed to classify different abnormalities in ECG signals (i.e., heartbeats) using CNNs.

As another approach, privacy and latency issues in edge learning were addressed by Dai et al. [63], in 2019, who proposed an on-device inference app for skin cancer detection. In their study, they suggested storing a pre-trained model on mobile devices for local inference, allowing patients to use the model to directly detect skin cancer from samples. This approach eliminates the need to send private data to the cloud and resolves latency issues.

## 8 Discussion

Decentralized machine learning involves deploying machine learning models in a decentralized manner, where data is distributed across nodes. There are two primary categories of decentralized learning systems. The first category includes systems with a centralized authority overseeing the learning process. In such setups, multiple nodes connect to a central server to collaboratively train machine learning models. Examples of these systems include federated learning, split learning, and edge learning, which are reviewed in this paper. Despite their advantages in facilitating collaboration while maintaining data privacy, such systems pose some challenges. Firstly, they establish a hierarchical power dynamic in which the central server assumes sole authority over the learning process, requiring trust from all involved parties. Additionally, this conventional client-server architecture shows low fault tolerance, as a server failure would result in the termination of the entire learning process [64]. Alternatively, in a peer-to-peer network, nodes communicate directly with each other, eliminating the need for a central server. This decentralization prevents the concentration of power and enhances fault tolerance, as unaffected nodes can continue training in the event of a node failure. Swarm learning and gossip learning exemplify such decentralized peer-to-peer systems.

Furthermore, these decentralized learning systems differ in terms of communication efficiency. For instance, split learning reduces the number of parameters that need to be sent in each communication round compared to federated learning. On the other hand, in a sequential split learning system, more communication rounds are needed as the number of clients increases. Similarly, peer-to-peer systems require more communication than centralized approaches, such as federated learning. In edge learning, edge servers help to reduce communication overhead compared to cloud servers since data and most communications stay on the edge of the network.

Regarding the computational power of devices in the network, in cross-silo settings, the entities are usually organizations or hospitals with reliable connections and strong computational power. Therefore, any technique, such as swarm learning, federated learning, and split learning, used in such settings is generally not limited by device power. However, in cross-device settings, which involve lightweight devices like cell phones or IoT devices, the limited computational power of participants becomes a critical consideration. In these scenarios, in the design of any of the decentralized systems, including federated learning, split learning, gossip learning, and edge learning, it is essential to have these constraints in mind.

Despite the variations in architectures, models, and implementations for decentralized privacy-preserving learning, regarding the prediction performance, it is commonly shown that decentralized learning approaches can achieve comparable performance to traditional machine learning implementations and to each other [65], [66], [67], [38], [26]. However, while decentralized machine learning models can prevent the direct sharing of raw data, they do not inherently guarantee privacy preservation, which can vary depending on the architecture and models used. Decentralized models can still leak sensitive information through model parameters, as they may reveal details about the training data [68]. The degree of privacy protection varies across algorithms, as it is harder, for instance, to reconstruct data from a deep neural network than from a linear model. Therefore, it is crucial to consider privacy risks when implementing decentralized algorithms and to employ robust security measures such as secure multi-party computation [69], homomorphic encryption [70], or differential privacy [71] to ensure data privacy. Multiple reviews and studies were published focusing on the attacks and defense mechanics of decentralized learning [72], [73], [74].

Lastly, it is important to clarify the terminology surrounding decentralized learning systems, as it can often be confusing or misleading. For example, the term decentralized federated learning is sometimes used to describe peer-to-peer learning systems. Similarly, edge-consensus learning [16], decentralized gossip mutual learning [17], or peer-to-peer learning [75] have been employed to refer to methods that are essentially variations of gossip learning. In non-medical contexts, peer-to-peer learning is traditionally used to refer to a decentralized system without a central server. These inconsistencies highlight the need for precise language to avoid confusion in the field.

## 9 Conclusion

Despite the various architectures and models available, decentralized privacy-preserving learning often matches the prediction performance of traditional machine learning and other decentralized methods. Therefore, we suggest selecting a suitable method based on factors such as the model, network capacity, network stability, computational power, and the desired level of control. For instance, a traditional federated learning approach is more appropriate when high control is desired, as the central server can govern the entire learning process. If the strategy relies on weak computational devices, edge learning can be considered. Swarm learning is ideal for stable networks with known participants. If network traffic is limited and a neural network is planned, split learning is a viable option. Nevertheless, these concepts can often be combined, allowing for hybrid architectures. The growing potential of these technologies to connect institutions highlights their significant role in the future of healthcare, especially in addressing challenges like the study of rare diseases and overcoming regional biases, by facilitating broader and more effective collaboration.

## CRediT authorship contribution statement

**Mohammad Tajabadi:** Writing – original draft, Methodology, Formal analysis, Conceptualization. **Roman Martin:** Writing – original draft, Methodology, Investigation, Conceptualization. **Dominik Heider:** Writing – review & editing, Supervision, Project administration, Funding acquisition, Conceptualization.

## Declaration of Competing Interest

Non declared.
